# Editorial Comment to A case of mixed tumor formed by metastasis of urothelial carcinoma and malignant lymphoma to the same lymph nodes

**DOI:** 10.1002/iju5.12343

**Published:** 2021-06-30

**Authors:** Takanori Sekito, Takuya Sadahira

**Affiliations:** ^1^ Department of Urology Dentistry and Pharmaceutical Sciences Okayama University Graduate School of Medicine Okayama Japan

The current article by Kobayashi *et al*. reported a rare case of a mixed tumor in a para‐aortic lymph node that comprised a metastatic urothelial carcinoma and Hodgkin lymphoma.^1^ To our best knowledge, the coexisting metastases of urothelial carcinoma and Hodgkin lymphoma in the same lymph node have never been reported. This article has potential importance for daily clinical practice.

We read the article with great interest because of the extremely rare phenomenon of a mixed tumor derived from different origins. Hypothetical mechanisms in the development of mixed tumors have been described.^2^ One important hypothesis concerns changes in the surrounding microenvironment induced by a primary tumor that lead to increased risk of a secondary tumor. Some cancer cells have also been reported to produce chemokines that induce lymphocytic migration.^3^ In the reported case, the pathogenesis of the composite tumor consisting of carcinoma and lymphoma could be attributed to both hypothetical mechanisms, although it is also possible that the mixed tumor occurred coincidentally.

Methotrexate (MTX)‐associated lymphoproliferative disorders (LPDs) can be considered in patients undergoing long‐term MTX treatment, because these patients have a relatively high risk of lymphoma.^4^ The patient in this case had chronic rheumatoid arthritis and had received MTX for more than 10 years. In cases of MTX‐associated LPDs, withdrawal of MTX can occasionally lead to spontaneous complete remission, as mentioned in previous reports.^5^ Hodgkin lymphoma in the reported case could have been an MTX‐associated LPD, and management of immunosuppressive treatment might have been be an important clinical factor.

## Conflict of interest

The authors declare no conflict of interest.
